# Human Leucocyte Antigen (HLA) B27 Allele Positivity Rate Among Patients Suffering From Spondyloarthritis and Its Distribution According to Age and Gender: A Hospital-Based Cross-Sectional Study

**DOI:** 10.7759/cureus.108503

**Published:** 2026-05-08

**Authors:** Himamoni Deka, Pankaj Kakati, Sultana J Ahmed, Kritanjali Dutta

**Affiliations:** 1 Anatomy, Gauhati Medical College and Hospital, Guwahati, IND; 2 Human Leukocyte Antigen (HLA) Laboratory, Gauhati Medical College and Hospital, Guwahati, IND; 3 Community Medicine, Assam Medical College, Dibrugarh, IND

**Keywords:** ankylosis spondylitis, autoimmune disease, hla-b27 allele, prevalence, spondyloarthritis

## Abstract

Introduction

The risk of developing spondyloarthritis (SpA) is significantly increased by human leucocyte antigen (HLA) B27 allele positivity. Despite the wide genetic diversity among the population of North-East India, studies on SpA and its genetic markers are scarce. The current study employed the sequence-specific primer polymerase chain reaction (SSP-PCR) technology to estimate the HLA-B27 allele positivity rate among patients suffering from SpA and to assess its distribution according to age and gender.

Methods

Ethylenediaminetetraacetic acid (EDTA) blood samples were collected from routine cases of clinically diagnosed/suspected SpA. DNA was isolated from all blood samples using a silica-based spin column approach. PCR amplification for the HLA-B27 allele was performed using the SSP technique.

Results

In this study, 207 clinically diagnosed/suspected SpA cases were tested for the HLA-B27 allele. Out of 207 cases, 40 (19.3%) had a clinical diagnosis and 167 (80.7%) had a clinical suspicion with a variety of symptoms. Of these, 70 (33.8%) patients had positive HLA-B27 allele tests, with 59 (35.5%) being clinically suspected. Age and gender were found to significantly affect HLA-B27 allele positivity, especially in cases that were clinically suspected (p<0.05). HLA-B27 allele positivity varied significantly with age and gender (p<0.05). The most often diagnosed clinical form of spondylitis was ankylosing spondylitis with 28 (70%) cases among whom seven (25%) were HLA-B27 allele positives. Backache and stiffness in the lower back and hip were the commonest presentation among clinically confirmed cases and were mostly observed among ankylosing spondylitis cases (24, 88.9%). For the clinically suspected cases, the most prevalent presenting symptoms were lower back pain (56, 33.5%) and back pain (33, 19.8%). It was discovered that HLA-B27 allele positivity was independently associated with male sex and people between 15 and 30 years of age.

Conclusion

HLA-B27 allele is prevalent in this region with male preponderance among the patients. HLA-B27-allele-positive patients might encounter an early onset of symptoms. The positivity rate was nearly twice as high in men and three times higher among individuals aged 15-30 years. Early diagnosis and understanding of disease patterns may enhance treatment effectiveness. Hence, it might be used for the early diagnosis of the disease.

## Introduction

Evidence connecting human leucocyte antigen (HLA) B27 allele to ankylosing spondylitis has existed since the early 1970s. This genotype is widespread in the general population, but it significantly increases the risk of developing spondyloarthritis, a connected family of inflammatory rheumatologic disorders [1]. The major histocompatibility complex class I of genes on chromosome 6 that are found on all nucleated cells contain the gene loci for the human leukocyte antigens (HLA) [2]. According to research on animals, the HLA-B27 allele by itself can cause a condition that resembles spondyloarthritis (SpA), which makes this allele unique. Although numerous research studies have examined the relationship between the HLA region and SpA, the precise mechanism by which HLA-B27 allele predisposes individuals to acquire the condition is still unknown [3].

The risk of developing SpA, which includes axial SpA, with or without peripheral arthritis, enthesitis, acute anterior uveitis, and gastrointestinal inflammation, is significantly increased by the HLA-B27 allele [4]. Apart from that, it is also found to be associated with diseases affecting different human organs and systems. Bakland et al. revealed that the high prevalence of HLA-B27 leads to a significant increase in the prevalence of axial SpA compared to radiologic SpA among chronic back pain patients [5]. According to a study by Ziade, Middle Eastern and Arab nations had substantially lower rates of HLA-B27 (0.3% to 6.8%) in the general population than Western nations (6% to 25%) [6]. Nevertheless, they proposed that HLA-B27 testing may be helpful in diagnosing axial SpA positivity. The presence of HLA-B27 in axial SpA patients was linked to male sex, younger age, longer disease duration, stronger family aggregation, and higher incidence of uveitis in a recent study conducted on the Chinese population [7]. According to Jayaprakash et al. (2022), men aged 16-25 years had a greater prevalence of HLA-B27 among SpA patients [8]. A strong association between HLA-B27 allele and axial SpA suggests that it might be frequently used for diagnosis [9]. The prompt identification of SpA may facilitate the start of efficient medical care, reducing the burden of disease and preventing expenses.

As young, productive adults are frequently impacted by SpAs, it has a significant socioeconomic effect and lowers the quality of life (QoL) of the affected individuals [10]. Because of its complex etiopathogenesis, the prevalence of SpA varies by region. The genetic architecture of North-East India is considerably more complicated than that of East and Southeast Asia, involving a combination of interactions between geography, ethnicity, and languages. The North-Eastern Indian state of Assam has an area of 78,438 km^2^. With almost 31 million residents, it is the largest state in terms of population and the second largest by land in North-Eastern India. There are 35 districts in the state classified into five divisions: Barak Valley, Central Assam, North Assam, Lower Assam, and Upper Assam. So far, there is a limited amount of literature and studies available on SpA and its associated genetic markers from this part of the country. Therefore, the present study aimed to use the sequence-specific primer polymerase chain reaction (SSP-PCR) technique to estimate HLA-B27 allele positivity rate among clinically diagnosed/suspected patients with different forms of SpA and its distribution in various age groups and genders. We combined and compared clinically diagnosed and suspected cases of SpA to identify patients with HLA B27 allele positivity, as it indicates genetic susceptibility of the disease.

## Materials and methods

**Figure 1:**
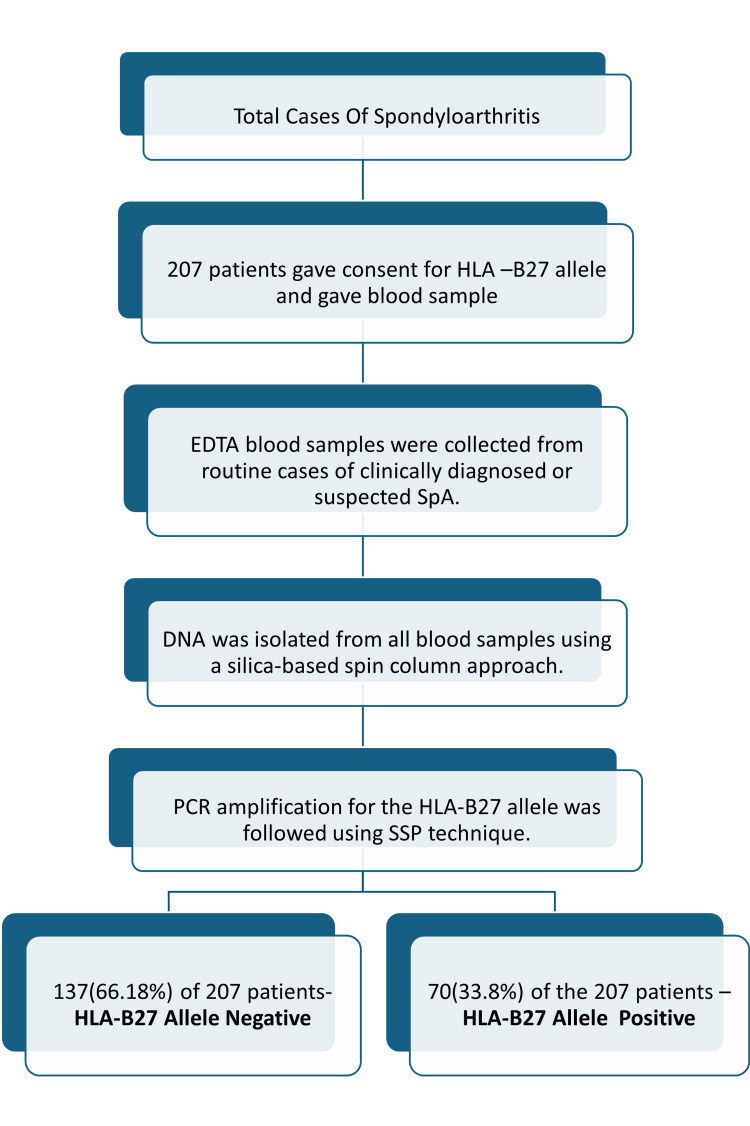
Methodology followed explained through a flowchart

The present study was a retrospective cross-sectional study conducted in the Human Leukocyte Antigen (HLA) Laboratory, Department of Anatomy, Gauhati Medical College, during March 2019 to January 2023. Routine cases of clinically diagnosed/suspected SpA from different outpatient and inpatient departments, viz., Orthopaedics, Neurology, and Medicine, of the study institution were included in the study. Patients from these departments who fulfilled the working definition were consecutively included in the study. Ethylenediaminetetraacetic acid (EDTA) blood samples were collected from routine cases of clinically diagnosed/suspected SpA and transported to the HLA Laboratory. Ethical Clearance for the study was obtained from the Institutional Ethics Committee (IEC) of the Gauhati Medical College, Guwahati. Informed consent was obtained from each participant for enrolling and collecting data for the study. Details of the socio-demographic profile of the participants, family history, and clinical symptoms were recorded on a pre-structured proforma. The study methodology is explained in Figure 1.

DNA was extracted from all the collected blood samples using silica-based spin column technique. The polymerase chain reaction (PCR) amplification for HLA-B27 allele was performed using SSP techniques. All the PCR reagents except molecular-grade water were provided in the Inno-Train Diagnostics HLA-Ready Gene B27 SSP kit (Inno-Train Diagnostik GmbH, Kronberg, Germany), which included tubes coated with primers: PCR buffer, Taq polymerase - 5U/µL, and 50 bp DNA ladder. For the PCR technique, the PCR programme shown in Table 1 was followed.

**Table 1:** Steps followed to perform polymerase chain reaction (PCR)

1x Initial	10x cycles	20x cycles	Hold
96°C for 2 min	96°C for 15 sec	96°C for 15 sec	4°C for infinity, or until post-PCR processing
	65°C for 60 sec	61°C for 50 sec	
		72°C for 30 sec	

**Figure 2:**
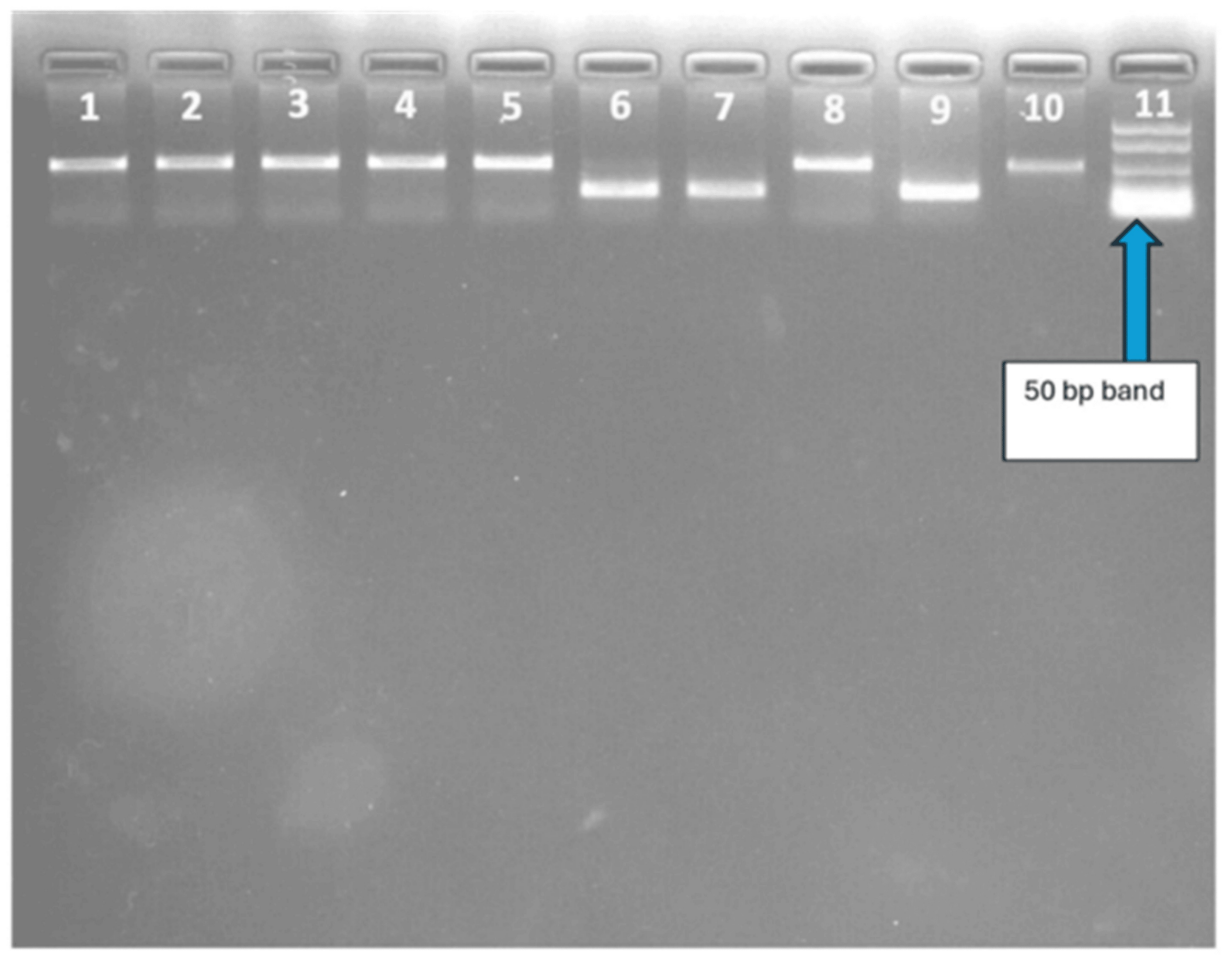
The PCR gel electrophoresis for HLA-B27 allele. Lane 11 represents the 50 bp ladder, Lane 10 represents negative control (the negative control used in the methods was the standard negative control provided within the kit used), Lane 9 represents positive control (the positive control used in the study was the standard positive control provided within the kit used). Lanes 1, 2, 3, 4, 5 and 8 represent negative HLA-B27 allele. Lanes 6 and 7 represents positive HLA-B27 allele.

PCR products were then loaded onto 2% agarose gel and gel electrophoresis procedure was carried out. Agarose gel was analyzed and interpreted using the gel documentation system. For quality control, an intrinsic control band of 430 bp was used along with a ladder of 50 bp during standardization. A negative control provided with the kit was used to detect contamination. A positive control was also used to assess the HLA-B27 allele positive. The PCR product of HLA-B27 allele, at 150 bp, was found in 2% agarose gel documentation system after electrophoresis. The PCR product of 150 bp was interpreted as positive for HLA-B27 allele. The absence of PCR product of 150 bp but presence of an intrinsic control band only at 430 bp was interpreted as negative for HLA-B27 allele (Figure 2).

The working definition for spondyloarthritis is as follows: clinically diagnosed case - if a patient fulfils three mentioned criteria: (a) low back pain (three months) and stiffness that improves with exercise but not by rest, (b) limitation of lumbar spine motion, and (c) limitation of chest expansion; the clinically suspected case - if a patient has low back pain (three months) and stiffness. The modified New York criteria for ankylosing spondylitis are presented in Table 2.

**Table 2:** Modified New York criteria for ankylosing spondylitis Classification: Definite ankylosing spondylitis: if one radiological criterion is associated with one clinical criterion. Probable ankylosing spondylitis: if the three clinical criteria are present without any radiologic criterion or if there is radiological criterion without any clinical criterion.

Clinical Criteria	Radiologic Criteria
		(a) Low back pain (3 months) and stiffness that improves with exercise but not by rest	(a) Sacroiliitis grade ≥2 bilaterally or sacroiliitis grade 3-4 unilaterally
(b) Limitation of lumbar spine motion	(b) Sacroiliitis grade 3-4 unilaterally
(c) Limitation of chest expansion	

The inclusion criteria are routine cases of clinically diagnosed/suspected SpA from different outpatient and inpatient departments, viz. Orthopaedics, Neurology, and Medicine, of the study institution. ​If the patients refused to take part in the study, they were excluded.

Statistical analysis

The data were analysed using Statistical Package for the Social Sciences (SPSS) version 21 (IBM Corp., Armonk, NY). Continuous variables were expressed as mean and standard deviation. On the other hand, categorical variables were presented using percentages and frequencies. Using the Kolmogorov-Smirnov test, the normality of continuous variables was examined. The chi-square test and the z-test for proportions were used to determine whether the proportional differences were statistically significant. Univariate and multivariate binary logistic regression models with backward elimination were employed to find independent factors linked to HLA-B27 positivity. A p-value was considered statistically significant if it was less than 0.05.

## Results

A total of 207 clinically diagnosed/suspected cases of SpA were tested for HLA-B27 allele positivity using the SSP-PCR technique in the current study. Out of them, 40 (19.3%) were clinically diagnosed cases with different forms of SpA, while 167 cases (80.7%) were clinically suspected cases with various symptoms. Out of the 207 cases, 70 (33.8%) tested positive for HLA-B27, of whom a majority (59, 35.5%) were clinically suspected cases. The mean age (±standard deviation) of HLA-B27-allele-positive cases was 32.36 (±13.82) years, which was significantly lower (p<0.05) than that of the HLA-B27-allele-negative cases (39.47±15.08). Also, in case of the clinically suspected cases, the mean age of the HLA-B27-allele-positive cases was found to be significantly lower as compared to the HLA-B27-allele-negative cases (p<0.001). However, no significant difference in mean age was noted in HLA-B27-allele-negative and -positive clinically diagnosed patients (Table 3).

**Table 3:** The age of patients tested for the presence of HLA-B27 allele The data were represented as mean and standard deviation. P<0.05 was considered significant for the t-test.

Age at presentation of cases	HLA-B27 allele	Mean age	Standard deviation	t-test	P-value
All cases (n=207)	Positive (n=70) (33.82%)	32.36	13.82	3.39	0.001
	Negative (n=137) (66.18%)	39.47	15.08		
Clinically diagnosed cases (n=40)	Positive (n=11) (27.5%)	37.36	15.47	0.19	0.84
	Negative (n=29) (72.5%)	38.41	14.57		
Clinically suspected cases (n=167)	Positive (n=59) (35.33%)	31.42	13.43	3.64	<0.001
	Negative (n=108) (64.67%)	39.75	15.27		

The gender distribution of the cases revealed a male preponderance with male:female ratio of 1.4:1. A significant difference (chi-square value=7.33; p<0.05) was noted in HLA-B27 allele positivity between the genders. Out of the total 70 HLA-B27-allele-positive cases, 50 (71.4%) were men. HLA-B27 allele positivity was also significantly different (chi-square value=4.38, p<0.05) between genders in clinically suspected cases (Table 4). 

**Table 4:** Distribution of HLA-B27 allele positivity status of the participants according to gender (total sample, N=207) M: Male; F: female.

Participant categories	Sex	HLA B27 allele		Chi-square value	P-value
		Negative	Positive		
All cases	Total (N=207)	137 (100.0%)	70 (100.0%)		
	F (n=86)	66 (48.2%)	20 (28.6%)	7.33	0.007
	M (n=121)	71 (51.8%)	50 (71.4%)		
Clinically diagnosed cases (n=40)	Total (N=40)	29 (100.0%)	11 (100.0%)		
	F (n=20)	17 (58.6%)	3 (27.3%)	3.13	0.08
	M (n=20)	12 (41.4%)	8 (72.7%)		
Clinically suspected cases (n=167)	Total (N=167)	108 (100.0%)	59 (100.0%)		
	F (n=66)	49 (45.4%)	17 (28.8%)	4.38	0.036
	M (n=101)	59 (54.6%)	42 (71.2%)		

**Figure 3:**
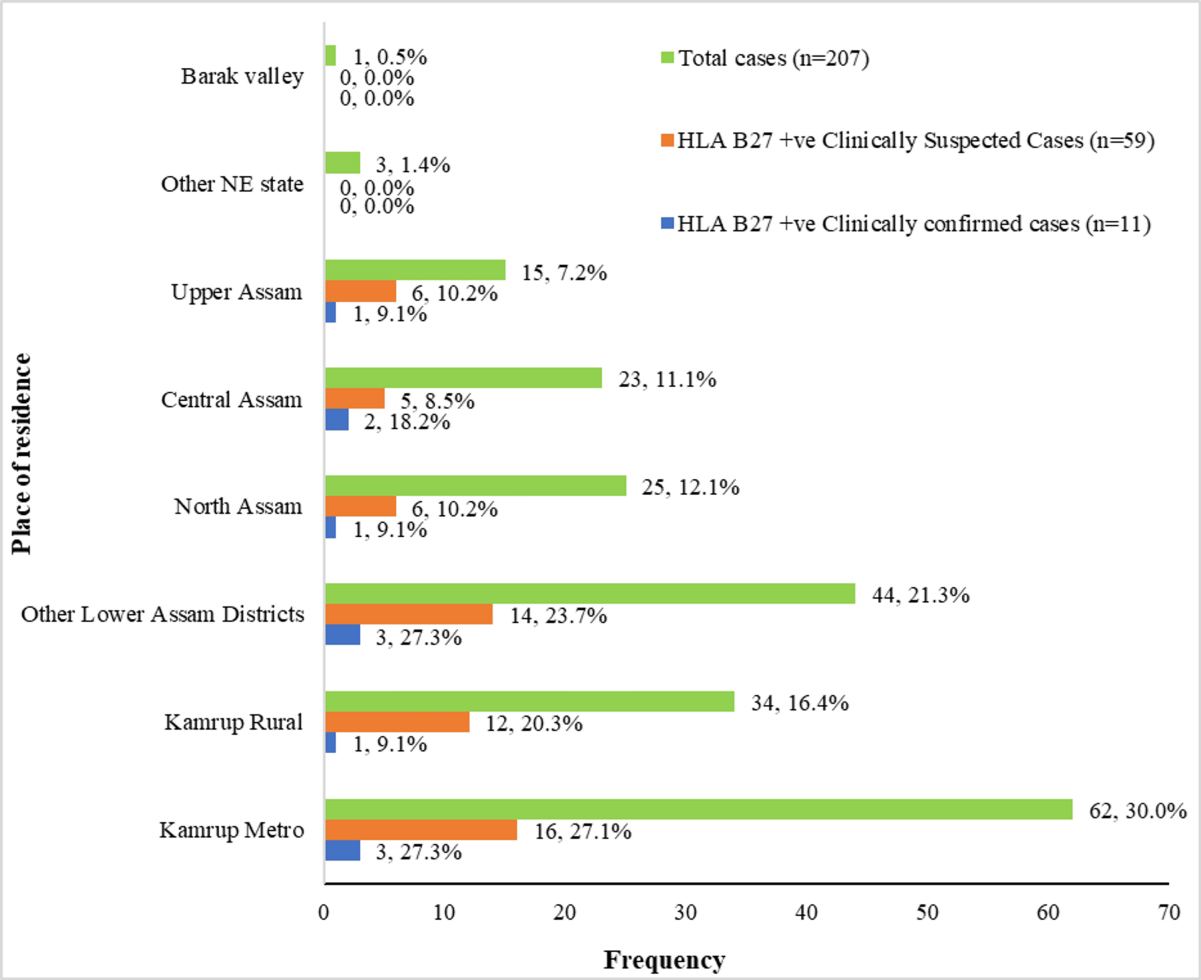
Distribution of total cases and HLA B27 allele positive cases by district in Assam Total cases (n=207) (green); HLA B27 allele +ve clinically suspected cases (n=59) (orange); HLA B27 allele +ve Clinically confirmed cases (n=11) (blue)

The majority of the participants were from Kamrup metro (n=62, 30.0%), Kamrup rural (n=34, 16.4%), and other lower Assam districts (n=44, 21.3%). Kamrup metro and other lower Assam districts (n=3, 27.3%) contributed the majority of HLA-B27-allele- positive clinically confirmed cases. While in case of clinically suspected HLA-B27-allele-positive cases, Kamrup metro (n=16, 27.1%) and rural districts (n=12, 20.3%) contributed the highest load (Figure 3).

Among the 40 clinically diagnosed cases, 28 (70%) were diagnosed with ankylosing spondylitis, six (15%) with psoriatic arthritis, and three (7.5%) with polyarthritis. Only seven out of 28 ankylosing spondylitis cases were HLA-B27 allele positive. Psoriatic arthritis, polyarthritis, cervical arthritis, and rheumatoid arthritis constituted the remaining four HLA-B27-allele-positive cases (Table 5).

**Table 5:** Type of arthritis in clinically diagnosed cases and HLA-B27 status (N=40) The data were represented as frequency (n) and percentage (%); a p-value <0.05 was considered for z-test.

Type of arthritis	HLA-B27		z-value	p-value
	Negative (n=29)	Positive (n=11)		
Ankylosing spondylitis (n=28)	21 (75.0%)	7 (25.0%)	0.54	0.59
Psoriatic arthritis (n=6)	5 (83.3%)	1 (16.7%)	0.64	0.52
Poly arthritis (n=3)	2 (66.7%)	1 (33.3%)	0.23	0.81
Osteo arthritis (n=1)	1 (100.0%)	0 (0.0%)	0.62	0.53
Cervical arthritis (n=1)	0 (0.0%)	1 (100.0%)	-1.64	0.10
Rheumatoid arthritis (n=1)	0 (0.0%)	1 (100.0%)	-1.64	0.10

Out of the 28 clinically confirmed ankylosing spondylitis cases, a majority (24, 88.9%) presented with back ache and stiffness in lower back and hip among whom six (25.0%) were HLA-B27 positive. All the six psoriatic arthritis cases were presented with skin rashes, joint pain and joint swelling. No significant differences were noted in HLA-B27 allele positivity within various clinical symptoms among different types of arthritis (Table 6).

**Table 6:** Clinical profile of clinically confirmed cases and HLA-B27 allele status (N=40) The data were represented as frequency (n) and percentage (%); a p-value <0.05 was considered for z-test.

Type of Arthritis	Symptoms	HLA-B27		z- value	P-value
		Negative (n=29)	Positive (n=11)		
Ankylosing spondylitis	Back ache, stiffness in lower back and hip (n=24)	18 (75.0%)	6 (25.0%)	0.43	0.67
	Backache, stiffness (n=3)	2 (66.7%)	1 (33.3%)	-0.23	0.81
	Lower back ache (n=1)	1 (100.0%)	0 (0.0%)	0.62	0.53
Psoriatic arthritis	Skin rashes, joint pain, joint swelling (n=6)	5 (83.3%)	1 (1.7%)	0.64	0.52
Osteoarthritis	Knee pain, swelling, stiffness (n=1)	1 (100.0%)	0 (0.0%)	0.62	0.53
Polyarthritis	Multiple joint pain, swelling (n=3)	2 (66.7%)	1 (33.3%)	-0.23	0.81
Cervical spondylitis	Neck pain, stiffness (n=1)	0 (0.0%)	1 (100.0%)	-1.64	0.10
Rheumatoid arthritis	Small joint pain, morning stiffness (n=1)	0 (0.0%)	1 (100.0%)	-1.64	0.10

Out of the 167 clinically suspected cases, majority (56, 19.8%) presented with lower back pain, followed by joint pain in 49 (29.3%) and back pain in 33 (19.8%) cases. HLA-B27 positivity was mostly noted among those who have joint pain with or without swelling. Out of 59 cases with joint pain, 25 (44.6%) were HLA-B27 positive. Additionally, HLA-B27 positivity was noted in seven out of 17 cases (41.2%) with whole-body pain. However, no significant difference was noted in HLA- B27 positivity within various clinical symptoms (Table 7).

**Table 7:** Clinical profile of clinically suspected cases and HLA-B27 status (N=167) The data were represented as frequency (n) and percentage (%); p<0.05 was considered for z-test.

Symptoms	HLA-B27		z- value	P-value
	Negative (n=108)	Positive (n=59)		
Whole body pain (n=17)	10 (58.8%)	7 (41.2%)	-0.53	0.59
Lower back pain (n=56)	41 (73.2%)	15 (26.8%)	1.64	0.10
Joint pain (n=49)	27 (55.1%)	22 (44.9%)	-1.67	0.09
Back pain (n=33)	23 (69.7%)	10 (30.3%)	0.67	0.50
Joint pain with swelling (n=7)	4 (57.1%)	3 (42.9%)	-0.42	0.67
Eye problem (n=2)	2 (100.0%)	0 (0.0%)	1.05	0.29
Neurological problem (n=3)	1 (33.3%)	2 (66.7%)	-1.14	0.25

The univariate logistic regression analysis revealed that HLA-B27 positivity was significantly almost three times higher in the 15-30 years age group (odds ratio (OR)=2.86; 95% confidence interval (CI): 1.33-6.16) as compared to individuals above 45 years of age. Also, the HLA positivity was almost twice in men compared to women (OR=2.32; 95% CI: 1.25-4.31). After adjusting for the type of cases, individuals in the 15- to 30-year age bracket and male sex were found to be independently associated with HLA-B27 positivity (Table 8).

**Table 8:** Logistic regression analysis for association of age, gender and type of patients with HLA-B27 positivity (N=207) OR: Odds ratio; CI: confidence interval.

Variables	Categories	HLA-B27		Crude OR	95% CI Lower-Upper	Adjusted OR	95% CI Lower-Upper
		Negative (n=137)	Positive (n=70)				
Type of cases	Confirmed	29 (72.5%)	11 (27.5%)	1 (ref)	-		
	Suspected	108 (64.7%)	59 (35.3%)	1.44	0.67-3.09		
Age group	≥ 45 years	48 (77.4%)	14 (22.6%)	1 (ref)	-	1 (ref)	-
	5-14 years	6 (54.5%)	5 (45.5%)	2.86	0.76-10.78	2.89	0.75-11.16
	15-30 years	36 (54.5%)	30 (45.5%)	2.86	1.33-6.16	2.63	1.20-5.72
	31-44 years	47 (69.1%)	21 (30.9%)	1.53	0.70-3.36	1.48	0.67-3.29
Sex	Female	66 (76.7%)	20 (23.3%)	1 (ref)	-	1 (ref)	-
	Male	71 (58.7%)	50 (41.3%)	2.32	1.25-4.31	2.19	1.16-4.10

## Discussion

SpA is a broad category of inflammatory rheumatic disorders with unique genetic and clinical traits. In most cases, patients are clinically presented with axial or peripheral SpA. After rheumatoid arthritis, SpA is the second most prevalent inflammatory arthritis in India with an estimated prevalence of seven to nine per 10,000 persons [11]. The identification of the HLA-B27 as a contributor to disease susceptibility confirmed the substantial hereditary propensity, which had been hypothesized in the 1970s [12]. In the present study, 207 clinically diagnosed/suspected cases of SpA were tested for HLA-B27 allele positivity using the SSP-PCR technique, out of which 40 (19.3%) had a clinical diagnosis of SpA in one of its various forms, and 167 (80.7%) had a clinical suspicion of SpA in a variety of symptoms.

The overall HLA-B27 positivity was 33.8% (70), of whom the majority (59, 35.5%) were clinically suspected cases. Out of 167 suspected cases, 59 (35.3%) cases were HLA allele positive and out of 40 diagnosed cases, 27.5% was HLA allele positive. A similar study from South India reported HLA-B27 positivity rate among SpA patients at 54% [8]. The average age (±standard deviation) of clinically diagnosed cases was 38.13±14.63 years, which agrees with another study [12]. The mean age was substantially lower (p<0.05) in HLA-B27-allele-positive individuals than that of HLA-B27-allele-negative cases, particularly among the clinically suspected cases. Various studies have also reported that HLA-B27 allele positivity is significantly associated with earlier disease onset and diagnosis [13,14]. The gender distribution of the cases revealed a male preponderance with a male:female ratio of 1.4:1, which is similar to another study [15]. A recent hospital-based study from North-East India also reported male preponderance in SpA with a male:female ratio of 3.2:1 [16]. Only 20 (23.2%) of the 86 females tested positive for HLA-B27 allele compared to 50 (41.3%) out of 121 males, thus showing a significant variation in HLA-B27 allele positivity between the genders (chi-square value=7.33; p<0.05). The variation was mostly noted in the clinically suspected cases (chi-square value 4.38; p<0.05). Early disease onset and more severe sacroiliitis were linked to male gender and positive HLA-B27 allele status in ankylosing spondylitis [17].

Among the 40 clinically diagnosed cases, majority (28, 70%) were diagnosed with ankylosing spondylitis. A previous study conducted by Malakar et al. at a tertiary care hospital in North-East India documented ankylosing spondylitis as the commonest subtype of SpA in this region, which is in accordance with the findings of the present study. In this study, the HLA-B27 test was done in 14 patients with ankylosing spondylitis. Among them, 10 (73.7%) patients were HLA-B27 positive [16]. However, the HLA-B27 positivity among ankylosing spondylitis cases in our study (25%, n=7) is quite lower than the above study. This discrepancy may be attributed to differences in sample size, population ethnicity, and flow of patients to the health institution. In ankylosing spondylitis patients with the HLA-B27 gene, clonally increased CD8+ T cells are observed in the inflammatory tissues and the bloodstream. Furthermore, these T-cell receptors' α and β chain motifs have a special affinity for specific peptides made by the self and by microorganisms, which sets off an autoimmune response that ultimately results in the onset of the illness [18]. Lower back pain with 56 (33.5%) cases, joint pain with 49 (29.3%) cases, and back pain with 33 (19.8%) cases were the most common presenting symptoms among the 167 clinically suspected cases. Axial spondyloarthritis (axSpA) still has an unacceptably long wait time between symptom onset and diagnosis [19]. Several studies have reported the presence of inflammatory back pain (IBP), one of the factors used to classify axial SpA, as a criterion for referral to a rheumatologist. Yet, considering the prevalent nature of back pain, it might be difficult to differentiate SpA from other causes of back pain due to IBP's low specificity for a SpA diagnosis [20].

As per the univariate logistic regression analysis, odds of HLA-B27 allele positivity were nearly three times higher in the 15- to 30-year age group (OR=2.86; 95% CI: 1.33-6.16) than in the over-45-year age group. Additionally, men had about twice as much likelihood of HLA allele positivity as women (OR=2.32; 95% CI: 1.25-4.31). Male sex and those aged 15-30 years were shown to be independently linked with HLA-B27 allele positivity in the multivariate model. Various studies have reported a greater proportion of HLA-B27 allele positives in the 15- to 30-year age bracket and in men, which agrees with our findings [21,22]. Thus, HLA-B27 allele positives might have an early diagnosis of SpA compared to the average age at clinical diagnosis [12], in agreement with the findings of Chaudhary et al. [22].

The Gauhati Medical College and Hospital is a pioneering tertiary care centre of Assam, situated in the Kamrup Metro district of Lower Assam. In the present study, the location-wise distribution of cases showed that Kamrup metro, Kamrup rural, and other Lower Assam districts contributed the highest SpA case load and HLA-B27-allele-positive cases.

Limitations

The present study was conducted at a single tertiary care centre. Since the present study was a time-bound single-centre study, it may not completely represent the true burden of the disease in the region. As it is a hospital-based cross-sectional study, patients coming to the hospital were enrolled. Patients in a hospital are not representative of the general population. Taking this as a baseline study, multicentric studies with a larger sample size may help in identifying the HLA-B27 allele status in SpA and its associated factors in the study region.

## Conclusions

HLA-B27 allele positivity is prevalent among individuals with SpA in the study region. Among those who tested positive for HLA-B27 allele, joint discomfort with or without swelling was a prevalent clinical complaint. HLA-B27 allele positivity was significantly associated with male gender and age. The positivity rate was nearly twice as high in males and three times higher among individuals aged 15-30 years. Early diagnosis and understanding of disease patterns may enhance treatment effectiveness. SpA may be diagnosed earlier in individuals who are HLA-B27 positive. However, multicentric cohort or case-control studies are needed to establish the strength of the association between HLA-B27 allele positivity and SpA. Additionally, a population-based cross-sectional study with a larger sample size would help estimate the prevalence of the HLA-B27 allele in the general population.
